# Energy Consumption Minimization in Unmanned Aerial Vehicle-Enabled Secure Wireless Sensor Networks

**DOI:** 10.3390/s23239411

**Published:** 2023-11-26

**Authors:** Xufei Ding, Wen Tian, Guangjie Liu, Xiaopeng Ji

**Affiliations:** School of Electronics and Information Engineering, Nanjing University of Information Science & Technology, Nanjing 210044, China; xufeiding@163.com (X.D.); csusttianwen@163.com (W.T.); gjieliu@gmail.com (G.L.)

**Keywords:** unmanned aerial vehicle (UAV), wireless sensor network (WSN), data collection, trajectory optimization, energy minimization

## Abstract

In wireless sensor networks (WSNs), unmanned aerial vehicles (UAVs) are considered an effective data collection tool. In this paper, we investigate the energy-efficient data collection problem in a UAV-enabled secure WSN without knowing the instantaneous channel state information of the eavesdropper (Eve). Specifically, the UAV collected the information from all the wireless sensors at the scheduled time and forward it to the fusion center while Eve tries to eavesdrop on this confidential information from the UAV. To surmount this intractable and convoluted mixed-integer non-convex problem, we propose an efficient iterative optimization algorithm using the block coordinate descent (BCD) method to minimize the maximum energy consumption of the ground sensor nodes (GSNs) under the constraints of secrecy outage probability (SOP), connection outage probability (COP), minimum secure data, information causality, and UAV trajectory. Numerical results demonstrate the superiority of the algorithm we proposed in energy consumption and secrecy rate compared with other schemes.

## 1. Introduction

Wireless sensor networks (WSNs), owing to their decentralized control and freeform arrangement, have become prevalent across various applications, including intelligent living, weather monitoring, and health tracking [1,2]. While in regions with sturdy network infrastructure, WSNs can effortlessly link up to the internet and transmit data to the collector [3], in far-flung and inconvenient areas like deserts and plateaus where base stations are not readily deployable, WSNs confront insurmountable hurdles in direct communication with the fusion center [4]. Against this backdrop, unmanned aerial vehicles (UAVs) are emerging as a feasible choice for mobile data collectors for WSNs. Thanks to their pliable deployment and user-friendly control, UAVs can effectively overcome the communication gap and provide a reliable mechanism for WSNs in remote locations [5]. In summary, wireless sensor networks, despite their many merits, are limited in their application in regions where network infrastructure is weak or nonexistent. Fortunately, the deployment of UAVs as mobile data collection tools for WSNs offers a solution to this challenge [6].

WSNs typically consist of a plethora of economical wireless ground sensor nodes (GSNs). In most research on secure sensor networks, there are few studies on the lifespan of UAV-assisted sensor networks. Most papers focus on the energy consumption or communication rate issues of UAVs. However, the energy consumption of these sensors poses a potential threat to the WSN’s lifespan [7]. To combat this issue, researchers have proposed a flexible trajectory design of UAVs, which incorporates a sleep and wake-up mechanism to efficiently gather information and preserve GSNs’ energy consumption [8]. To further elucidate, the sleep and wake-up mechanism in WSNs implies that when the GSN is not engaged in any communication with the UAV, it goes into a state of dormancy to conserve energy. Conversely, when the UAV approaches the GSN, the GSN promptly awakens and begins transmitting information to the UAV. However, given the constant movement of the UAV, it is essential to consider the highly dynamic wireless channels between the UAV and the GSNs to avoid any unexpected packet loss [9]. Therefore, reasonable UAV trajectory planning is an indispensable factor that must be taken into account [10].

In addition, the advent of UAVs has made wireless communications a breeze, thanks to their superior information transmission rates [11] and reduced transmit delay [12]. However, their broadcast characteristics make them a susceptible target for illegal eavesdroppers (Eve) [13]. Fortunately, physical layer security, a promising secure communication technology that is extensively employed, plays a pivotal role in preventing the prying eyes of Eve [14]. But here is the catch: in practice, it is very ideal to assume that the channel state information (CSI) of Eve is completely known [15]. Therefore, it is meaningful to discuss UAV-assisted secure communication when the CSI of Eve is unknown.

Driven by the aforementioned facts and challenges, we discuss a UAV-assisted secure WSN to reduce energy consumption in sensor networks while considering the unknown CSI of Eve in secure communication. Considering that turning off the sensors when they are not working can save a lot of energy, we introduce the sleep and wake-up mechanism to reduce sensors’ energy consumption through trajectory planning of the UAV. In this paper, we aim to minimize the maximum energy consumption of the GSNs when sending covert information by jointly optimizing the GSN scheduling and the trajectory of the UAV. Moreover, we take things a step further and elevate the discourse by acknowledging the harsh realities of practical scenarios, wherein the instantaneous CSI of Eve is far from perfectly known. The main contributions of this paper are summarized as follows.

1. The crux of our proposition involves an adaptive secrecy transmission policy, which is centered around the classic Wyner encoding scheme. Considering the instantaneous CSI of the Eve link is unknown, we derive an expression for confidentiality capacity under the connection outage probability (COP) and the secrecy outage probability (SOP) constraints.
2. We formulate the energy optimization problem of GSNs as a joint optimization problem that includes GSN scheduling and UAV trajectory. The optimization is subject to several constraints, including COP, SOP, minimum secure communication requirements, GSN scheduling, and UAV trajectory. By solving this problem, we aim to minimize energy consumption and maximize the secrecy rate as much as possible through trajectory optimization while satisfying the aforementioned constraints.
3. We put forward an iterative optimization algorithm based on the block coordinate descent (BCD) approach to transform the intractable optimization problem into two subproblems: GSN scheduling and the UAV trajectory. In the final stage, the optimization problem is solved by alternating iterative optimization of GSN scheduling and UAV trajectory. It is worth mentioning that our algorithm is ultimately convergent, a property that has been mathematically proven.

The rest of this paper is organized as follows. Section 2 introduces the related research about the UAV-enabled secure WSN. Section 3 has a detailed account of the system model. Section 4 and Section 5 establish an optimization problem of energy consumption minimization and propose an iterative optimization algorithm to solve it. Furthermore, the effectiveness of the proposed algorithm is verified in Section 6. Finally, the conclusion of this paper is in Section 7.

## 2. Related Work

### 2.1. The Application of UAVs in Secure WSNs

UAVs have a wide application space in WSNs, which cannot directly communicate with the data center. Ref. [16] discuss a UAV-powered WSN, where the UAV transmits energy to the ground sensor through the antenna, and the sensor will send the collected information to the UAV after receiving it. The author minimizes the time required for the UAV to collect information by jointly optimizing the height of the UAV and the antenna beamwidth. In [17], the authors proposed a task offloading mechanism learning algorithm, which can predict the queuing delay of all UAVs, reduce network overhead and increase user satisfaction. Ref. [18] considered a large-scale WSN where some GSN may not be able to upload information for a long time, resulting in insufficient storage capacity. The authors proposed a data collection strategy to minimize the data loss by jointly optimizing the sensor scheduling and the UAV’s trajectory. Refs. [19,20] investigated the energy consumption problem of the UAV-assisted WSN. Zhu et al. [19] proposed a novel optimization algorithm based on a deep reinforcement learning technique that can effectively reduce the UAV’s consumption. Beak et al. [20] model the UAV collecting ground sensor information as a non-convex problem, and optimize the trajectory by the Voronoi diagram to maximize the residual energy after the sensor transmits information.

### 2.2. Security Performance in UAV-Enabled WSNs

Since UAVs are more vulnerable to eavesdropping by illegal parties, some recent studies have considered the physical layer security of UAV-assisted WSNs. Ref. [21] investigate a UAV-assisted WSN with multiple eavesdroppers, and considered a downlink secure transmission scheme based on power splitting, where the transmission power of the UAV is divided into information transmission and noise generation. The authors proposed an optimization algorithm to maximize the minimum average secrecy rate. In [22], the authors considered how to improve the quality of service (QoS) of the wireless networks, joint optimization of the video levels selection, power allocation, and a UAV trajectory algorithm is proposed to maximize the ratio of power consumption to video quality. Refs. [23,24] discussed secrecy capacity maximum problem in cache-enabled UAV communications. Ref. [23] investigate a UAV-enabled network with D2D communications, where the UAV and D2D transmitter are equipped with caches that the users can directly obtain high-frequency communication requirements without communicating with the base station. In [24], the caching-equipped UAV is used to replace the small cell to communicate with the user, and the replaced cell is used as the interference source to send interference signals to Eve to improve the security performance of the system.

### 2.3. Secrecy Energy Efficiency in UAV-Enabled WSN

To realize the goal of energy-efficient communication while ensuring communication, secrecy energy efficiency (SEE) has increasingly become hot research in UAV-assisted WSNs. Li et al. [25] discuss two main challenges in a UAV-enabled WSN: the UAV’s energy consumption and secure transmission. The authors proposed a low-complexity iterative algorithm to maximize the secrecy energy efficiency. In [26], the authors discussed a multi-carrier multi-UAV enabled WSN, where the UAVs use Cooperative Rate-Splitting (CRS) technique to protect the communication between UAVs and the ground sensors, and proposed a secure resource allocation alternating iterative algorithm to maximize the UAV’s SEE by jointly optimizing the resource allocation and the ground sensors’ association matrix. Refs. [27,28] both introduced the simultaneous wireless information and power transfer (SWIPT) technology when considering the maximization of Secrecy Energy Efficiency, among which Ref. [27] assumed that the users divide the received signal into two parts, which are used for energy collection and information decoding, respectively. Ref. [28] assumed that only known the channel distribution information (CDI) of Eves. In addition, the dual-layer PS receiver architecture is introduced to solve the problem of energy harvesting (EH) circuits’ performance limitation.

## 3. System Model

**Figure 1:**
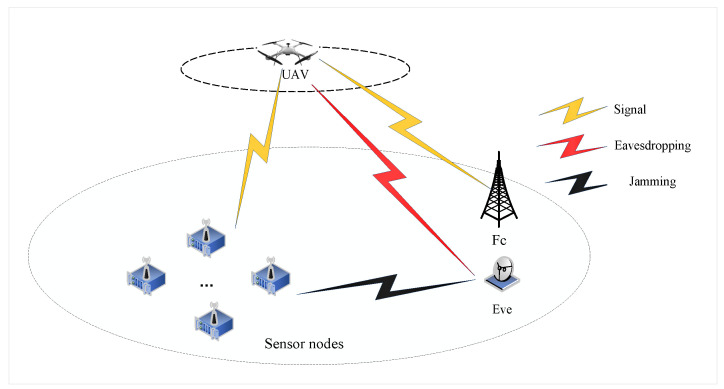
System model.

As shown in Figure 1, we consider a UAV-enabled secure WSN where a UAV of FD model is employed as a covert collector to receive the confidential information sent by the ground sensors and transmit the information to the fusion center (FC). The information here can include local communication, logistics, weather, etc. To facilitate subsequent data processing and without significant loss of generality, we assume that the whole model is based on a three-dimensional (3D) Cartesian coordinate system. We assume that there are *M* GSNs denoted by M={1,2,…,m,…M} and all GSNs equipped with an antenna to send collected information to the UAV. We assumed that the UAV’s fly altitude *H* is fixed, and $V_{max}$ represents the maximum flight speed. In addition, the start and end locations, which are denoted as $q_{u}^{0}=\left\{x_{0},y_{0},H\right\}$ and $q_{u}^{N}=\left\{x_{N},y_{N},H\right\}$, respectively, are also pre-determined. The total time required for the UAV to perform the task is *T*. We decompose the time *T* into *N* parts, N={1,2,…n,…,N}, and the length of each time gap is θ, i.e., T=θN. The UAV’s coordinate at the time slot *n* is $q_{u}\left[n\right]=\left\{x_{u}\left[n\right],y_{u}\left[n\right],H\right\}$, and the coordinates of the SNs, Eve and the FC are denoted as $q_{m}=\left\{x_{m},y_{m},0\right\}$, $q_{e}=\left\{x_{e},y_{e},0\right\}$ and $q_{f}=\left\{x_{f},y_{f},0\right\}$, respectively. We have the UAV’s trajectory and start/end location constraints:

(1) $$\begin{array}{c} ∥q_{u}\left[n\right]-q_{u}\left[n-1\right]∥\leq V_{max}\theta ,\forall n\geq 2\\ q_{u}\left[1\right]=q_{u}^{0},q_{u}\left[N\right]=q_{u}^{N} \end{array}$$

We tend to adopt the Line of Sight (LoS) channel model for the UAV to GSNs links during this paper. This is often a reasonable assumption, since some researchers have proved that when UAVs fly at a sufficiently high altitude, the LoS channel dominates the UAV-to-ground channel [29]. Thus, the channel power gain from GSNs to the UAV, the UAV to the FC, and Eve can be expressed as:

(2) $$\begin{array}{c} g_{u,m}\left[n\right]=\beta_{0}d_{u,m}^{-\zeta }\left[n\right]=\frac{\beta_{0}}{{||q_{u}\left[n\right]-q_{m}||}^{2}+H^{2}},\forall m,\\ g_{u,f}\left[n\right]=\beta_{0}d_{u,f}^{-\zeta }\left[n\right]=\frac{\beta_{0}}{{||q_{u}\left[n\right]-q_{f}||}^{2}+H^{2}}\\ g_{u,e}\left[n\right]=\beta_{0}d_{u,e}^{-\zeta }\left[n\right]=\frac{\beta_{0}}{{||q_{u}\left[n\right]-q_{e}||}^{2}+H^{2}} \end{array}$$

where ζ=2 denotes the path-loss exponent and $\beta_{0}$ represents the reference channel gain at l=1m.

In addition, the channel power gain from GSNs to Eve can also be expressed as consisting of small-scale fading and large-scale path loss and can be given by

(3) $$\begin{array}{c} g_{m,e}=\beta_{0}{∥q_{m}-q_{e}∥}^{-\zeta }\varphi \end{array}$$

where φ represents the Rayleigh fading obeying exponential distribution with unit mean.

We assume that the wake-up and data-transmission policy is employed since the ground nodes’ power is limited [30]. Specifically, the UAV can control whether the ground nodes wake up. Only when the UAV wakes up the GSNs can the information transmission between them be carried out. At this time, other nodes are in the shutdown state. Moreover, the communication models between GSNs and the UAV are adopted the periodic time-division multiple access (TDMA) manner. In other words, the UAV can only transmit with one GSN at the same time slot. This can not only save energy consumption of GSNs but also avoid mutual interference between ground nodes during information transmission.

Define the binary wake-up scheduling variables $w_{m}\left[n\right]\in \left\{0,1\right\}$ at time slot *n*. When $w_{m}\left[n\right]=1$ if the ground node sends data to the UAV; otherwise, $w_{m}\left[n\right]=0$. Since only one device and the UAV have the communication link at time slot *n*, the user scheduling constraints can be expressed as follows:

(4) $$\begin{array}{c} \sum_{m=1}^{M}w_{m}\left[n\right]\leq 1,\forall n\in N,w_{m}\left[n\right]\in \left\{0,1\right\}. \end{array}$$

Denoting the transmit power of the UAV and the GSNs as $P_{u}$ and $P_{m}$. Due to the UAV’s hardware limitations, although many self-interference (SI) cancellation technologies proposed, the UAV self-interference still exists. Let ω represent the level of SI cancellation. At the time slot *n*, when the GSNs are in the wake-up state for transmitting data to the UAV, i.e., $w_{m}\left[n\right]=1$, the channel capacity between the UAV and GSNs can be expressed as:

(5) $$\begin{array}{c} C_{u,m}\left[n\right]=\log_{2}\left(1+\frac{P_{u}g_{u,m}\left[n\right]}{P_{u}{\left|g_{u,u}\left[n\right]\right|}^{2}+\sigma^{2}}\right),\forall m, \end{array}$$

where the $\sigma^{2}$ denotes the noise power, and the UAV’s self-interference $g_{u,u}$ follows CN(0,ω), ω is the UAV’s self-interference level. Similarly, the channel capacity between the UAV and Eve at the time slot *n* can be expressed as

(6) $$\begin{array}{c} C_{u,e}\left[n\right]=\log_{2}\left(1+\frac{P_{u}g_{u,e}\left[n\right]}{P_{m}g_{d,e}+\sigma^{2}}\right) \end{array}$$

## 4. Problem Formulation

Since Eve’s CSI is unknown, we introduce the classic Wyner’s secrecy encoding scheme [31], where named two rates: the codeword rate $R_{u,m}\left[n\right]$ representing the size of the transmitted code word and the secrecy rate $R_{sec}\left[n\right]$. The information rate of Eve represents $R_{u,e}\left[n\right]$, and the secrecy rate can be expressed as:

(7) $$\begin{array}{c} R_{sec}\left[n\right]={\left[R_{u,f}\left[n\right]-R_{u,e}\left[n\right]\right]}^{+}. \end{array}$$

where $R_{u,f}\left[n\right]$ represent the FC’s throughput.

Since there exists the inference channel, the codeword rate $R_{u,m}\left[n\right]$ between the UAV and the fusion center may be higher than the channel capacity $C_{u,m}\left[n\right]$, resulting the communication links to be interrupted. The COP denoted by $p_{m}^{cout}\left[n\right]$ represents the situation happening can be expressed as

(8) $$\begin{array}{cc} \; & p_{m}^{cout}\left[n\right]=Pr\left(C_{u,m}\left[n\right]<R_{u,m}\left[n\right]\right)\\ \; & =\mathrm{Pr}\left(\log_{2}\left(1+\frac{P_{m}g_{u,m}\left[n\right]}{P_{u}{\left|g_{u,u}\left[n\right]\right|}^{2}+\sigma^{2}}\right)<R_{u,m}\left[n\right]\right)\\ \; & =\mathrm{Pr}\left(|g_{u,u}{\left[n\right]|}^{2}>\frac{1}{P_{u}}\left(\frac{P_{m}g_{u,m}\left[n\right]}{2^{R_{u,m}\left[n\right]}-1}-\sigma^{2}\right)\right)\\ \; & =1-\mathrm{Pr}\left(|g_{u,u}{\left[n\right]|}^{2}\leq \frac{1}{P_{u}}\left(\frac{P_{m}g_{u,m}\left[n\right]}{2^{R_{u,m}\left[n\right]}-1}-\sigma^{2}\right)\right) \end{array}$$

which the PDF of $|g_{uu}{\left[n\right]|}^{2}$ obeys an exponential distribution with parameter 1/ω. So, the $p_{d}^{cout}\left[n\right]$ can be expressed as

(9) $$\begin{array}{cc} \; & p_{d}^{cout}\left[n\right]=\exp\left(-\frac{1}{P_{u}\omega }\left(\frac{P_{m}g_{u,m}\left[n\right]}{2^{R_{u,m}\left[n\right]}-1}-\sigma^{2}\right)\right) \end{array}$$

In addition, since the UAV cannot know the CSI of Eve, which means we do not have an ideal secure communication environment, and there may happen a secrecy outage incident. $p_{d}^{sout}$ represent the probability of this situation happening and can be expressed as

(10) $$\begin{array}{cc} p_{d}^{sout}\left[n\right] & =\mathrm{Pr}(C_{u,e}\left[n\right]>R_{u,e}\left[n\right])\\ \; & =\mathrm{Pr}\left(\log_{2}\left(1+\frac{P_{u}g_{u,e}\left[n\right]}{P_{m}g_{m,e}+\sigma^{2}}\right)>R_{u,e}\left[n\right]\right)\\ \; & =\mathrm{Pr}\left(g_{m,e}<\frac{1}{P_{m}}\left(\frac{P_{u}g_{u,e}\left[n\right]}{2^{R_{u,e}\left[n\right]}-1}-\sigma^{2}\right)\right) \end{array}$$

The PDF of $g_{m,e}$ obeys an exponential distribution function with parameter $1/(\beta_{0}d_{m,e}^{-\zeta })$ in (3), so the SOP can be given by

(11) $$\begin{array}{cc} \; & p_{d}^{sout}\left[n\right]=1-\exp\left(-\frac{1}{P_{m}\beta_{0}d_{m,e}^{-\zeta }}\left(\frac{P_{u}g_{u,e}\left[n\right]}{2^{R_{u,e}\left[n\right]}-1}-\sigma^{2}\right)\right). \end{array}$$

Whether the connection outage events or the secrecy outage events, it is something we do not want to happen. Assume the maximum terminal COP and SOP that we can tolerate are $\phi_{cout}$ and $\phi_{sout}$, respectively. So, we have the following constraints:

(12) $$\begin{array}{c} \sum_{m=1}^{M}w_{m}\left[n\right]P_{m}^{cout}\left[n\right]\leq \phi_{cout},\forall n,\sum_{m=1}^{M}w_{m}\left[n\right]P_{m}^{sout}\left[n\right]\leq \phi_{sout},\forall n \end{array}$$

Let $W=\{w_{m}\left[n\right]\},\forall d,n$, and $Q=\{q_{u}\left[n\right]\},\forall n$. Our goal is to minimize the maximum energy consumption of the GSNs when the UAV transmits covert signals. Mathematically, the energy-efficient data collection problem can be formulated as follows:

(13a) $$\begin{array}{c} \min_{\left\{W,Q\right\}}E_{max} \end{array}$$

(13b) $$\begin{array}{cc} s.t. & \sum_{n=1}^{N}w_{m}\left[n\right]E_{m}\leq E_{min},\forall m, \end{array}$$

(13c) $$\begin{array}{cc} \; & \sum_{m=1}^{M}w_{m}\left[n\right]P_{m}^{cout}\left[n\right]\leq \phi_{cout},\forall n, \end{array}$$

(13d) $$\begin{array}{cc} \; & \sum_{m=1}^{M}w_{m}\left[n\right]P_{m}^{sout}\left[n\right]\leq \phi_{sout},\forall n, \end{array}$$

(13e) $$\begin{array}{cc} \; & \sum_{n=1}^{N}w_{m}\left[n\right]\left(R_{u,f}\left[n\right]-R_{u,e}\left[n\right]\right)\geq \xi ,\forall m, \end{array}$$

(13f) $$\begin{array}{cc} \; & \sum_{n=1}^{N}w_{m}\left[n\right]R_{u,m}\left[n\right]\leq \sum_{n=1}^{N}w_{m}\left[n\right]R_{u,f}\left[n\right],\forall m, \end{array}$$

(13g) $$\begin{array}{cc} \; & \sum_{m=1}^{M}w_{m}\left[n\right]\leq 1,\forall n,w_{m}\left[n\right]\in \left\{0,1\right\}, \end{array}$$

(13h) $$\begin{array}{cc} \; & ∥q_{u}\left[n\right]-q_{u}\left[n-1\right]∥\leq V_{max}\theta ,\forall n\geq 2, \end{array}$$

(13i) $$\begin{array}{cc} \; & q_{u}\left[1\right]=q_{u}^{0},q_{u}\left[N\right]=q_{u}^{N}. \end{array}$$

where $E_{m}=P_{m}\theta$ is the energy consumption of the GSN in a one-time slot. Equations (13b) and (13d) are the COP and SOP constraint. *B* is the system bandwidth. ξ is the minimum confidential capacity we can receive. Equation (13f) is the information causality constraint which means the UAV cannot transmit the information that has not been received. Thus, the UAV’s throughput $R_{u,m}\left[n\right]$ should no more than the FC’s throughput $R_{u,f}\left[n\right]=\log_{2}\left(1+P_{u}g_{u,f}\left[n\right]/\sigma^{2}\right)$. Since we want the UAV can collect covert information as much as possible, there must have lower bounds of the COP and SOP. Equation (13f) is the GSN scheduling constraint. Equations (13h) and (13i) are the UAV’s mobile constraints. Obviously, the problem (13) is nonconvex, because of the complex constraints. Particularly, the constraints (13d) and (13e) are expressed in the form of probability, which makes it difficult for us to deal with this optimization problem.

Note that $p_{m}^{cout}\left[n\right]$ and $p_{d}^{sout}\left[n\right]$ are non-decreasing functions of $R_{u,d}\left[n\right]$ and $R_{u,e}\left[n\right]$, and it can be seen from (7) that when $R_{u,d}\left[n\right]$ increases, the security capacity of the UAV also increases. On the contrary, when $R_{u,e}\left[n\right]$ decreases, the security capacity also decreases. So, if we want to maximize the UAV’s secrecy rate, the outage probability must be minimum, and the constraint (12) should become equation form, i.e., $\sum_{m=1}^{M}w_{m}\left[n\right]P_{m}^{cout}\left[n\right]=\phi_{cout},\forall n;\sum_{m=1}^{M}w_{m}\left[n\right]P_{m}^{sout}\left[n\right]=\phi_{sout},\forall n$. In addition, since the UAV can only have communication with one ground node at one time slot, we can also have $P_{m}^{cout}=\phi_{cout}$ and $P_{m}^{sout}=\phi_{sout}$. Combing (8) and (10) and the above analysis, the throughput of the FC and Eve can be expressed as

(14) $$\begin{array}{cc} \; & R_{u,m}\left[n\right]\\ \; & =\log_{2}\left(1+\frac{P_{m}g_{u,m}\left[n\right]}{-P_{u}\omega ln\left(\phi_{cout}\right)+\sigma^{2}}\right)\\ \; & R_{u,e}\left[n\right]\\ \; & =\log_{2}\left(1+\frac{P_{u}g_{u,e}\left[n\right]}{-P_{m}\beta_{0}d_{d,e}^{-\zeta }\ln\left(1-\phi_{sout}\right)+\sigma^{2}}\right) \end{array}$$

In the sequel, after the above analysis and transformation, we can substitute Formula (14) into the problem (13) and remove the COP and SOP constraints, i.e., Equations (13b) and (13d). The optimization problem (13) can be simplified as follows:

(15) $$\begin{array}{cc} \; & \min_{\left\{W,Q\right\}}E_{max}\\ s.t.\;(13b),\;(13e),\;(13f), & (13g),\;(13h),\;(13i), \end{array}$$

Although we simplify the original optimization problem and remove the complex constraints of probability representation, problem (15) is still non-convex because of the existence of $R_{sec}\left[n\right]$. In addition, since the ground nodes’ scheduling variables are binary, the optimization problem (15) is nonconvex mixed-integer programming which is hard to cope with directly. In the sequel, we develop an iteration algorithm based on the block coordinate descent (BCD) method and the successive convex approximation (SCA) method to solve it.

## 5. Problem Solution

In this section, we split the original problem into two sub-problems based on the BCD method. In subproblem 1, with the given trajectory, we use the relaxation method to optimize the GSN schedulings. Subproblem 2 of optimizing the UAV’s trajectory with given GSN schedulings is solved by the SCA method.

### 5.1. The Optimization of GSN Scheduling

First, we optimize the ground nodes’ schedulings with the given UAV’s trajectory. To this end, by relaxing the binary constraints in problem (15), the standard linear program (LP) can be reformulated as:

(16) $$\begin{array}{cc} \; & \min_{\left\{W,Q\right\}}E_{max}\\ s.t.\;(13b),\;(13c), & (13f),\;(13h),\;(13i), \end{array}$$

where $R_{u,d}\left[n\right]$ and $R_{u,e}\left[n\right]$ can be obtained from (14). It is clear that (16) is an integer programming problem, which can be solved optimally with existing convex optimization techniques.

Notably, the optimization problem (16)’s optimal solution *W* is continuous. To convert the optimization results into the binary results we need, the rounding method in [32] is employed to cope with it. According to [32], this method not only does not affect the optimality, but can also effectively obtain the binary results we need through reconstructing the continuous results.

### 5.2. UAV Trajectory Optimization

Then, with the given scheduling *W*, the original optimization problem has been transformed into a problem of how to optimize the UAV’s trajectory to maximize the minimum secrecy capacity of all the SNs, so that we can not only minimize the energy consumption of the GSNs but also maximize the secrecy capacity as much as possible. Introducing the slack variable ϖ and $Q_{e}\left[n\right]$, the UAV’s trajectory optimization problem can be expressed as:

(17a) $$\begin{array}{cc} \; & \max_{\left\{Q,Q_{e},\varpi \right\}}\varpi \end{array}$$

(17b) $$\begin{array}{cc} s.t. & \sum_{n=1}^{N}w_{m}\left[n\right]\left(R_{u,f}\left[n\right]-R_{u,e}\left[n\right]\right)\geq \varpi ,\forall m, \end{array}$$

(17c) $$\begin{array}{cc} \; & \varpi \leq \sum_{n=1}^{N}R_{u,m}\left[n\right]\forall m, \end{array}$$

(17d) $$\begin{array}{cc} \; & ∥q_{u}\left[n\right]-q_{u}\left[n-1\right]∥\leq V_{max}\theta ,\forall n\geq 2, \end{array}$$

(17e) $$\begin{array}{cc} \; & q_{u}\left[1\right]=q_{u}^{0},q_{u}\left[N\right]=q_{u}^{N}. \end{array}$$

(17f) $$\begin{array}{cc} \; & Q_{e}\left[n\right]\leq {∥q_{u}\left[n\right]-q_{e}∥}^{2}, \end{array}$$

where

(18) $$\begin{array}{cc} \; & R_{u,m}\left[n\right]=\log_{2}\left(1+\frac{h}{H^{2}+{∥q_{u}\left[n\right]-q_{m}∥}^{2}}\right)\\ \; & R_{u,e}\left[n\right]=\log_{2}\left(1+\frac{P_{u}\beta_{0}}{gQ_{e}\left[n\right]}\right),\\ \; & R_{u,f}\left[n\right]=\log_{2}\left(1+\frac{P_{m}\beta_{0}}{\sigma^{2}(H^{2}+∥q_{u}\left[n\right]-q_{f}{∥}^{2})}\right), \end{array}$$

where $h=\frac{P_{u}\beta_{0}}{-P_{u}\omega \ln\left(\phi_{cout}\right)+\sigma^{2}}$, $g=-P_{m}\beta_{0}d_{m,e}^{-\zeta }\ln\phi_{sout}+\sigma^{2}$. Then, we introduce the following Lemma to deal with the nonconvex constraints (17b).

**Lemma** **1.** *The constraint (17b) can be rewritten by the following convex constraints.*

(19) $$\begin{array}{c} \sum_{n=1}^{N}w_{m}\left[n\right]\left(R_{u,f}^{lb}\left[n\right]-R_{u,e}\left[n\right]\right)\geq \varpi ,\forall m, \end{array}$$

*where*

(20) $$\begin{array}{cc} \; & R_{u,f}^{lb}\left[n\right]=\\ \; & \Phi^{l}\left[n\right]-\Theta^{l}\left[n\right]\left(∥q_{u}\left[n\right]-q_{f}{∥}^{2}-{∥q_{u}^{l}\left[n\right]-q_{f}∥}^{2}\right),\\ \; & \Theta^{l}\left[n\right]=\\ \; & \frac{P_{m}\beta_{0}}{\ln2(\sigma^{2}(H^{2}+∥q_{u}^{l}\left[n\right]-q_{f}{∥}^{2})+P_{m}\beta_{0})}\\ \; & \times \frac{1}{\sigma^{2}(H^{2}+∥q_{u}^{l}\left[n\right]-q_{f}{∥}^{2})},\\ \; & \Phi^{l}\left[n\right]=\log_{2}\left(1+\frac{P_{m}\beta_{0}}{\sigma^{2}(H^{2}+∥q_{u}^{l}\left[n\right]-q_{f}{∥}^{2})}\right), \end{array}$$

**Proof.**  Obviously, $R_{u,f}\left[n\right]$ is not a convex function about $q_{u}\left[n\right]$, but we can see that $R_{u,f}\left[n\right]$ is a convex function about $∥q_{u}\left[n\right]-q_{f}{∥}^{2}$. It is well known that the lower bound of any convex function at its feasible point can be obtained by its first-order Taylor transformation. Assuming that $Q^{l}\left[n\right]=\left\{q_{u}^{l}\left[n\right],\forall n\right\}$ represents the UAV’s trajectory optimization results at the *l*-th iteration, then at the feasible point $q_{u}^{l}\left[n\right]$, we can obtain the next iteration of $R_{u,f}\left[n\right]$:

(21) $$\begin{array}{cc} \; & R_{u,f}\left[n\right]\geq \\ \; & \Phi^{l}\left[n\right]-\Theta^{l}\left[n\right]\left(∥q_{u}\left[n\right]-q_{f}{∥}^{2}-{∥q_{u}^{l}\left[n\right]-q_{f}∥}^{2}\right)\\ \; & ≜R_{u,f}^{lb}\left[n\right] \end{array}$$

Similarly, we define $\Gamma_{e}\left[n\right]={\left(x_{u}\left[n\right]-x_{e}\right)}^{2}+{\left(y_{u}\left[n\right]-y_{e}\right)}^{2}+H^{2}$, $\Gamma_{m}\left[n\right]={\left(x_{u}\left[n\right]-x_{m}\right)}^{2}+{\left(y_{u}\left[n\right]-y_{m}\right)}^{2}+H^{2}$. By introducing the first-order Taylor transformation of Γ[n], we have the lower bounds of $\Gamma_{e}\left[n\right]$, $\Gamma_{m}\left[n\right]$ and $R_{u,m}\left[n\right]$:

(22) $$\begin{array}{cc} \; & \Gamma {\left[n\right]}_{e}\geq \Gamma_{e}^{l}\left[n\right]+2\left(x_{u}^{l}\left[n\right]-x_{e}\right)\left(x_{u}\left[n\right]-x_{u}^{l}\left[n\right]\right)\\ \; & +2\left(y_{u}^{l}\left[n\right]-y_{e}\right)\left(y_{u}\left[n\right]-y_{u}^{l}\left[n\right]\right)≜\Gamma_{e}^{lb}\left[n\right],\\ \; & R_{u,m}\left[n\right]\geq R_{u,m}^{lb}\left[n\right]=\log_{2}\left(1+\frac{h}{H^{2}+{∥q_{u}^{l}\left[n\right]-q_{m}∥}^{2}}\right)\\ \; & -\frac{h\left(∥q_{u}\left[n\right]-q_{m}{∥}^{2}-{∥q_{u}^{l}\left[n\right]-q_{m}∥}^{2}\right)\log_{2}e}{(h+H^{2}+∥q_{u}^{l}\left[n\right]-q_{m}{∥}^{2})(H^{2}+∥q_{u}^{l}\left[n\right]-q_{m}{∥}^{2})} \end{array}$$

where $\left(x_{u}^{l}\left[n\right],y_{u}^{l}\left[n\right]\right)$ represents the *l*-th iteration results of UAV’s trajectory.

As such, based on the above-mentioned results, the optimization problem can be approximated into the following convex problem:

(23) $$\begin{array}{cc} \; & \max_{\left\{Q,Q_{e},\varpi \right\}}\varpi \\ s.t. & \sum_{n=1}^{N}w_{m}\left[n\right]\left(R_{u,f}^{lb}\left[n\right]-\log_{2}\left(1+\frac{P_{u}\beta_{0}}{gQ_{e}\left[n\right]}\right)\right)\\ \; & \geq \varpi ,\forall d,\\ \; & \varpi \leq \sum_{n}^{N}R_{u,m}^{lb}\left[n\right],Q_{e}\left[n\right]\leq \Gamma_{e}^{lb}\left[n\right],\forall n,m,\\ \; & (13h),\;(13i). \end{array}$$

Observing that problem (23) is a convex problem, we can use the existing software tools such as CVX 2.2 to solve it efficiently. Thus, the optimization problem (17) can be solved by solving optimization problem (23) and constantly updating the feasible points $\left(x_{u}^{l}\left[n\right],y_{u}^{l}\left[n\right]\right)$. The details of the UAV’s trajectory optimization are shown in Algorithm 1. In addition, Algorithm 1’s objective values are non-decreasing and bounded during the iteration process, so Algorithm 1 is convergent. In the sequel, we introduce the overall algorithm to tackle the integer-relaxed problem (13).
**Algorithm 1** Successive convex optimization algorithm for Problem (23)1:Initialize iterations l=0 and trajectory of the UAV as ${\left\{x\left[i\right],y\left[i\right]\right\}}^{0}$;2:**Repeat**3:          Optimizing the problem (23) with given $Q^{l}\left[n\right]$ and $w_{m}\left[n\right]$, and obtain the optimal selection ${\left\{x_{u}\left[n\right],y_{u}\left[n\right]\right\}}^{*}$.4:         Update $Q^{l+1}\left[n\right]$ = ${\left\{x_{u}\left[n\right],y_{u}\left[n\right]\right\}}^{*}$.5:**Until** meeting the terminal condition6:**Return** the optimal trajectory ${\left\{x_{u}\left[n\right],y_{u}\left[n\right]\right\}}^{*}$=$Q^{l}\left[n\right]$
 □

### 5.3. Overall Iterative Algorithm and Convergence Analysis

According to the previous analysis, we first employ the BCD method to decompose the original optimization problem that is difficult to be solved directly into two sub-problems, then solve them separately, and finally jointly optimize the problem through the iterative algorithm. The iterative algorithm for the problem (15) is summarized in Algorithm 2.
**Algorithm 2** Overall alternating iterative algorithm1:Initialize iterations l=0, $w_{m}{\left[n\right]}^{0}$ and ${\left\{x\left[n\right],y\left[n\right]\right\}}^{0}$;2:**Repeat**3:          Optimizing the problem (16) with given $Q^{l}\left[n\right]$, and obtain the optimal selection $w_{m}^{l}\left[n\right]$.4:          Optimizing the problem (23) with given $Q^{l}\left[n\right]$ and $w_{m}^{l}\left[n\right]$, and obtain the optimal selection $Q^{l+1}\left[n\right]$.5:         Update l=l+16:**Until** meeting the terminal condition.

Then, we analyze the convergence of the algorithm. As presented, the Algorithm enables the satisfaction of constraints (13e) with equality, which is evident after step 4. Subsequently, due to the maximization of the weighted minimum throughput in (17), it is possible to relax constraints (13e) after step 4. This relaxation leads to an expanded optimization space that can be utilized for the reduction of $E_{max}$ in (16). Consequently, with Algorithm 2, the cost values obtained from (16) exhibit a non-increasing behavior across iterations. It is essential to note that the objective value of (16) can be lower-bounded by a finite value, thereby ensuring Algorithm 2’s convergence. Moreover, the computational complexity of Algorithm 2 mainly comes from steps 3 and 4, i.e., the optimization of the GSN schedulings and UAV’s trajectory. The proposed algorithm’s computational complexity is given by $O(IN^{\left(2\right)})$, where *N* is the number of time slots, and *I* is the number of iterations of Algorithm 2. As a result, the algorithm may be calculated in polynomial time, making it simple to implement in WSNs with limited resources.

## 6. Simulation Results

In this section, we verify the performance of the algorithm proposed through numerical simulations. The numerical results obtained by Matlab (2022b). The configuration of the computer is INTEL Core I9 13900KS and NVIDIA GeForce RTX 4090. The start/end locations of the UAV and the FC are all presented as (800,0,0). There are 4 GSNs located at (0,1000,0) m, (0,−1000,0) m, (1000,0,0) m, and (−1000,0,0) m, respectively. The detailed parameters are given in Table 1.

**Figure 2:**
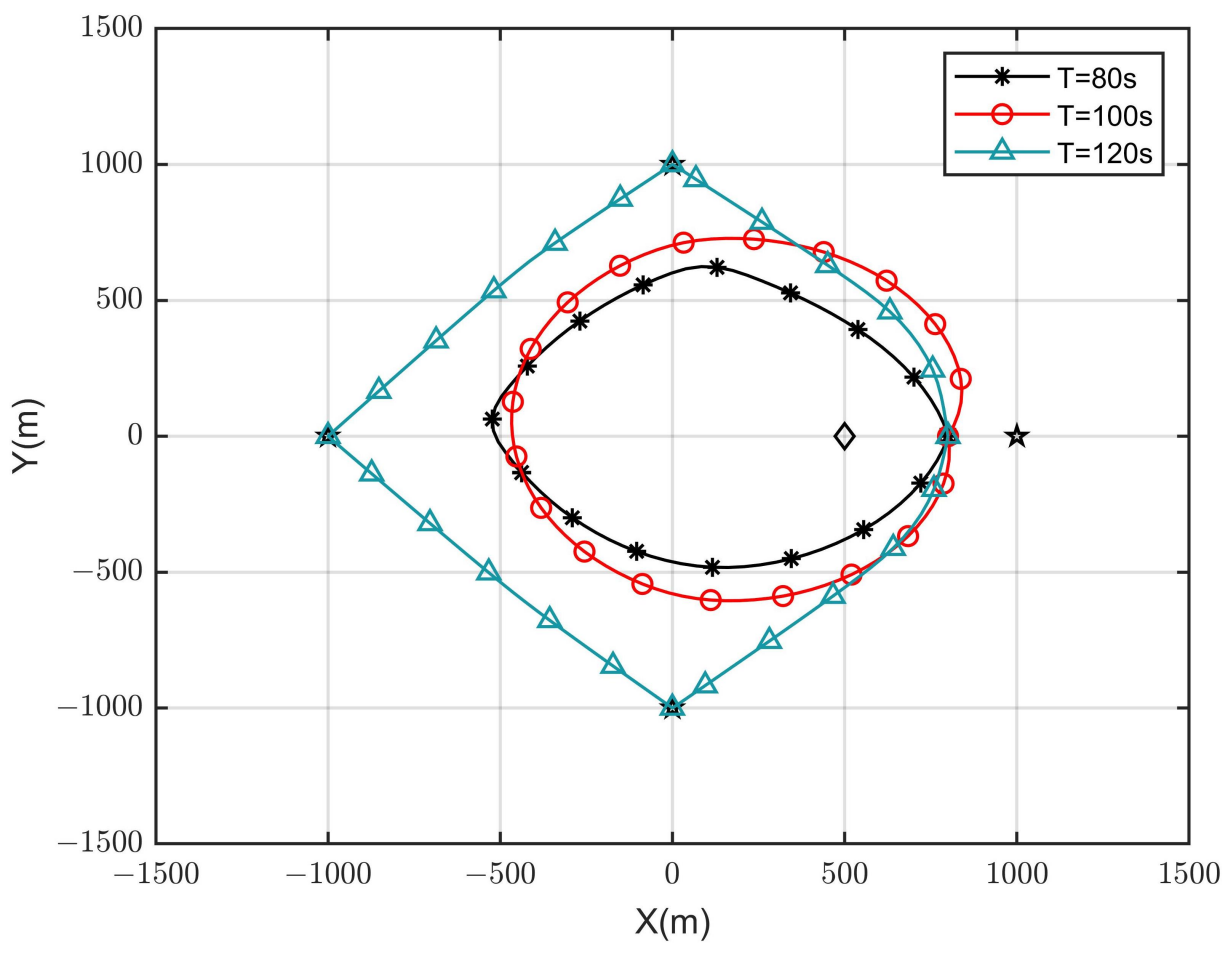
Optimized UAV trajectory in different values of *T*.

**Figure 3:**
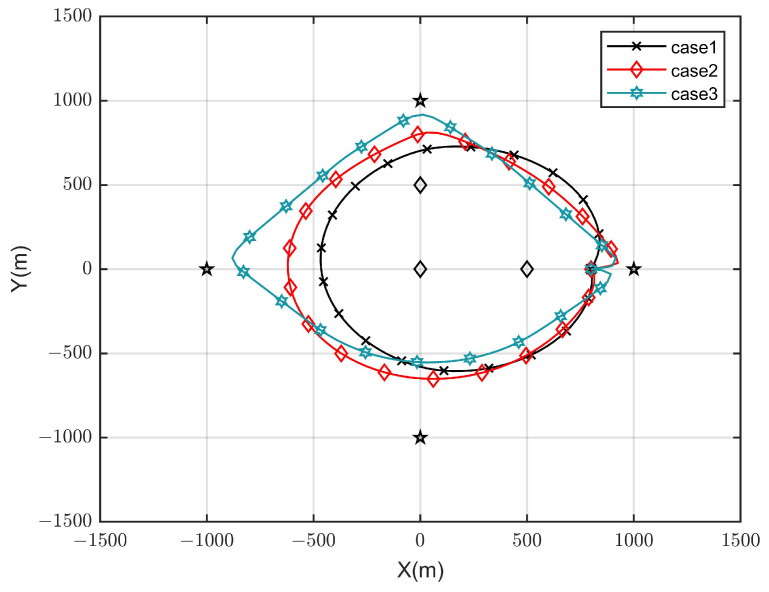
Optimized UAV trajectory under different cases.

**Figure 4:**
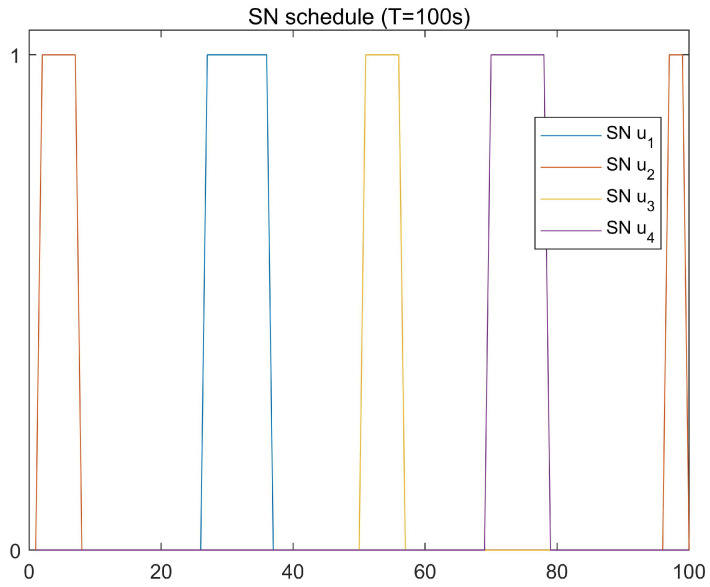
Optimized GSN scheduling.

The optimal UAV trajectories achieved by the proposed algorithm under different time *T* are shown in Figure 2, and Figure 3 shows the optimal UAV trajectories under case 1: $q_{e}=\left(500,0,0\right)$ m, case 2: $q_{e}=\left(0,0,0\right)$ m, and case 3: $q_{e}=\left(0,500,0\right)$ m with T=100 s. According to the results, when the time is enough, the UAV will always be as close to each ground GSN as possible in different cases to obtain a better channel state. At the same time, when approaching the Eve gradually, the UAV will stay away from the Eve while approaching the SN to obtain more secrecy capacity. Figure 4 shows the scheduling optimization results of SNs in case 2. It can be seen from the figure that the GSN is always in the closed state when it is not communicating with UAV, and only when the UAV is fully closed can it be in the communication state. This avoids interference with other SNs and fully saves energy when transmitting confidential information.

**Figure 5:**
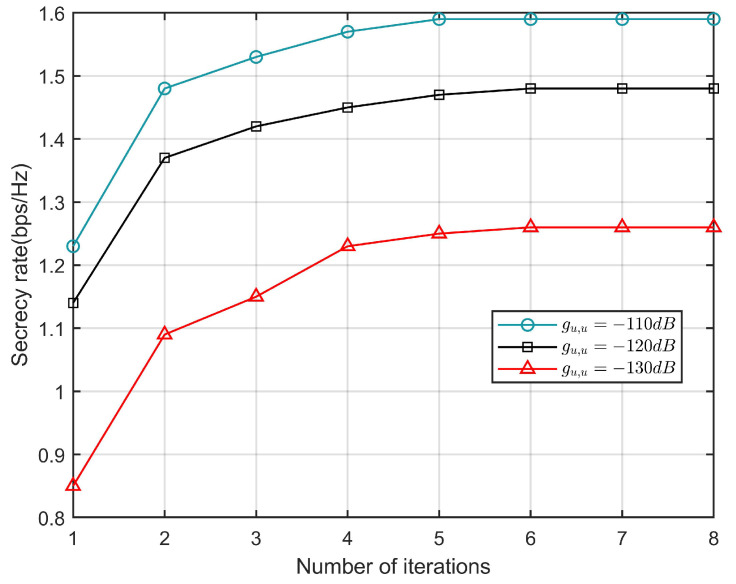
The convergence of the algorithm under different values of $g_{uu}$.

Figure 5 shows the convergence of the proposed iterative algorithm under different levels of UAV self-interference. It can be seen that with the increasing number of iterations, the secrecy rate of the UAV is non-decreasing, which is consistent with the convergence analysis results above. In addition, with the increasing degree of self-interference, the secrecy rate will also decrease. This is because with the increase in self-interference, the interference of the signal received by the UAV will be greater and the safety rate will be reduced.

**Figure 6:**
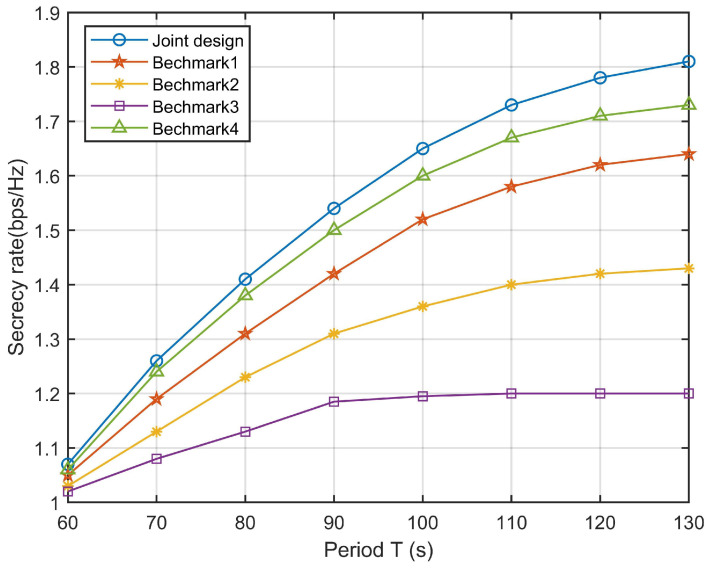
Secrecy rate comparison for different schemes and different values of *T*.

To better observe the effectiveness of the proposed algorithm, three benchmark schemes are considered for comparison: 1. Elliptic trajectory design with transmission power control. 2. Elliptic trajectory design with fixed transmission power. 3. UAV flies on a circular path with a radius of 500 centered on (0,0). 4. The UAV operating in TDD mode. Figure 6 shows the comparison of secrecy rate between our joint design scheme and other benchmark schemes at different times *T*, where Eve is at (0,500,0) m. It can be seen that with the increase in time *T*, the secrecy rate of the UAV is also increasing, because, with the increase in time, the UAV can stay more time at the ground SNs to obtain better channel status and collect more information. In addition, the performance of the scheme with trajectory optimization is always better than that without trajectory optimization, which indicates that trajectory optimization is important to improving the secrecy rate.

**Figure 7:**
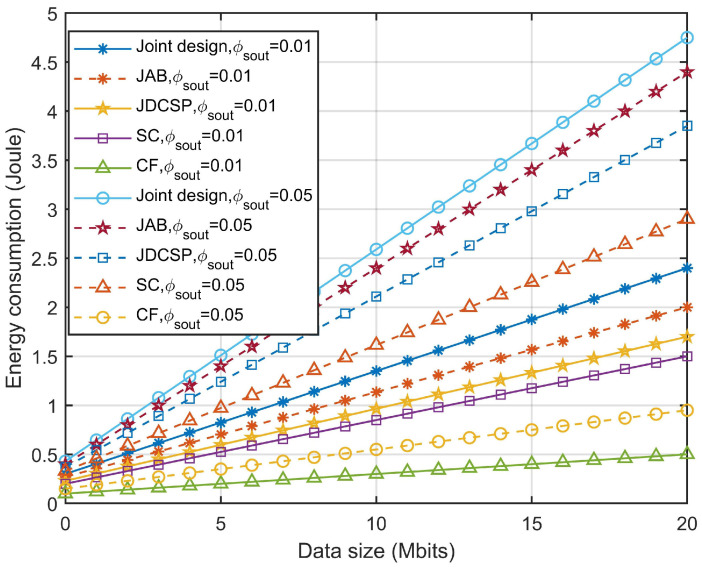
Energy consumption comparison for different schemes and different values of $\phi_{scout}$.

Figure 7 shows the energy consumption level of the proposed algorithm compared with the JAB algorithm [16], JDCSP algorithm [35], and other schemes under different SOP values. The SC and CF represent the static collecting scheme and the circular flight scheme [36], respectively. In Figure 7, it is evident that the proposed algorithm uses the least amount of energy, which indicates the importance of joint consideration of GSN scheduling and UAV trajectory. In addition, as SOP increases, the minimum energy consumption will as well, which is because the more stringent SOP requirements will cause the limited communication resources to be unable to support and make the energy consumption higher.

## 7. Conclusions

This paper studies an energy-efficient data collection scheme in a UAV-assisted secure WSN that employs a wake-up mechanism to effectively preserve the energy of SNs. To minimize the energy consumption of sensors while ensuring communication requirements, we proposed a joint optimization algorithm that first decomposes the original problem into two sub-problems based on the BCD method and iteratively tackle each sub-problem to achieve the overall optimization objective. The simulation results demonstrate the effectiveness of our proposed algorithm. Under the premise of meeting communication requirements, the reasonable trajectory planning of the UAV and the wake-up mechanism effectively reduce the transmission energy consumption of sensors. In future work, more channel models and communication technologies will be studied, such as the small-scale fading and the intelligent reflecting surface technology. In addition, better optimization methods that can reduce the algorithm complexity are expected.

## Figures and Tables

**Table 1:** Simulation parameters.

Parameters	Values
The altitude of the UAV [32], *H*	100 m
The reference channel power gain [32], $\beta_{0}$	−60 dB
The level of the UAV’s self-inference [15], ω	−120 dB
The noise power [26], $\sigma^{2}$	−110 dB
The maximum speed of the UAV [32], $V_{max}$	50 m/s
The length of each time slot [26], θ	0.5 s
The minimum tolerable data received [23], ξ,	100 Kbit
The power of the UAV and ground nodes [33], $P_{u},P_{m}$,	10 dBm
The maximum SOP\COP constraint [34], $\phi_{sout}\backslash \phi_{cout}$,	0.05

## Data Availability

Data are contained within the article.
